# Gastrointestinal Tract Polyp Detection, Tracking, and Surveillance and the Role of Artificial Intelligence

**DOI:** 10.7759/cureus.110074

**Published:** 2026-06-01

**Authors:** John Azizian, Bhaskar Banerjee, Oleh Haluszka, Rolando Leal

**Affiliations:** 1 Gastroenterology and Hepatology, University of Arizona College of Medicine - Tucson, Tucson, USA

**Keywords:** adenoma, artificial intelligence (ai), colonoscopy, colorectal cancer, esophagogastroduodenoscopy (egd), polyp, screening, surveillance

## Abstract

Colorectal cancer remains a common malignancy and a leading cause of cancer-related mortality in the United States. Colonoscopy is widely utilized for screening, with the goal of early detection of malignancy, as well as identification and removal of pre-cancerous lesions. Screening strategies are guided by individual risk factors, including family history, the presence of hereditary conditions associated with increased colorectal cancer risk, and findings from prior colonoscopic examinations, including polyp number, size, and histologic characteristics. These factors inform recommendations on the appropriate timing for initiation and surveillance intervals. Endoscopic resection is generally preferred for the removal of adenomatous polyps when feasible, while more advanced lesions may require surgical intervention or specialized endoscopic techniques. Despite advances in technique and operator experience, lesions may still be missed during colonoscopy. To address this limitation and increase the yield of colonoscopies detecting lesions, artificial intelligence (AI)-based tools have been increasingly incorporated to enhance polyp detection and improve overall procedural quality. These technologies aim to support endoscopists in identifying subtle lesions, thus increasing malignancy and polyp detection rates, and thereby potentially contributing to more effective colorectal cancer prevention, lowering healthcare costs, and ultimately improving overall patient outcomes.

## Introduction and background

A polyp in the gastrointestinal tract is an abnormal proliferation, or growth, of cells, usually forming a clump but varying in size and shape, which may be benign, pre-cancerous/neoplastic, or cancerous [1]. Examples of polyp shapes include sessile (flatter on the luminal lining), pedunculated (attached to a thinner piece of tissue called a stalk, oftentimes from stool rubbing on the polyp and causing the underlying mucosal layer to stretch at the base of the polyp), semi-pedunculated, fungating, and carpet-like (completely flat) [1-7].

The gold standard for colonic polyp detection and tracking is a high-quality colonoscopy [1,2]. Polyps are differentiated from healthy, normal tissue around them by various morphological changes that we will describe in the following section [1,7]. Colon polyps generally do not cause symptoms, but sometimes, if large, they can be obstructive and result in constipation or cause a gastrointestinal frank bleed that could possibly lead to anemia [7]. More importantly, they are encountered during screening and surveillance colonoscopies, with the aim of resecting pre-cancerous polyps before they can become malignant [7].

Even for the most experienced endoscopist, polyps can be missed during endoscopic examinations. It is in this sphere that artificial intelligence (AI) software and innovations may aid in polyp detection and subsequent surveillance. This narrative review is intended to provide a broad discussion of topics for gastroenterologists and fellows alike. Herein, we will describe the different kinds of polyps present in the colon, national guidelines and recommendations for conducting screening and surveillance colonoscopies based on the type and number of polyps encountered, and finally, the evolving role of AI in improving colorectal cancer screening and prevention outcomes.

## Review

Types of polyps

Hyperplastic polyps are commonly occurring benign, non-neoplastic polyps that are generally small lumps of tissue, often pale or similar in color to normal mucosal tissue [1]. They are more likely to be found in the left colon (sigmoid/rectosigmoid) [1]. These polyps are non-dysplastic, exhibit normal proliferative behavior, and do not possess abnormal cellular components microscopically or endoscopically [2].

Multiple studies, including one by Laiyemo et al., have shown that hyperplastic polyps in the left colon that are smaller than 1 cm in size do not tend to have a high risk for transformation into cancerous lesions [2]. However, other studies have suggested that right colon hyperplastic polyps may portend a slightly higher likelihood of finding a neoplastic polyp elsewhere in the colon, although it is debated to what degree the said probability is increased [3].

Inflammatory polyps are benign, atypically shaped areas of normal colonic mucosa that is regenerating from a localized bowel inflammation, erosion, or ulceration. These can often be seen in the setting of inflammatory bowel disease, but do not have any reported risk for malignant transformation, and oftentimes resolve on their own [4].

Sessile serrated adenomas are characteristically flat and tend to have an overlying mucus or muco-fecal cap. They classically appear to have a jagged or sawtooth appearance endoscopically and have a relatively high rate of displaying high-grade dysplasia on pathologic evaluation of tissue [5,6]. Thus, sessile serrated adenomas carry an increased risk of malignant transformation.

Traditional serrated adenomas differ in that they have a higher risk for developing into malignancy or having high-grade dysplasia in the future, and they are morphologically different from sessile serrated adenomas. While both have a characteristic sawtooth appearance, traditional serrated adenomas have pathologic findings of ectopic crypts, pseudostratification, and a villous pattern with stretched, elongated nuclei [7].

Non-serrated adenomatous polyps are the most common type of colonic polyp (comprising ~2/3 of polyps encountered on colonoscopy) and are pre-cancerous [8]. Their prevalence increases with age, and they are more common in men than in women [8]. Obesity also carries a heightened risk of developing adenomatous polyps, which is why it is suspected that there has been a progressive rise in adenoma detection rate in the United States as the incidence of obesity and average body mass index of the population also continues to increase [9]. While adenomatous polyps rarely have high-grade dysplasia, those that are 10 mm or larger in size can have as high as a 20%-30% chance of undergoing malignant transformation [10,11].

Non-serrated adenomatous polyps are divided further into three subtypes based on morphology and pathology: tubular, villous, and tubulovillous. Tubular adenomas are the most common type of adenomatous polyps and have a branching net-like appearance of the epithelial tissue that forms the polyp [12]. Villous adenomas classically contain long, thin finger-like glands that converge toward the center of the polyp [12]. Tubulovillous adenomas have intermixed morphological features of both tubular and villous adenomas [12]. Advanced adenomas are defined as polyps that are larger than 10 mm in size, exhibit high-grade dysplasia, and are of the tubulovillous or villous subtypes [13].

High-grade dysplasia is a midpoint in the progression from low-grade dysplasia to malignancy. Polyps with this designation do not extend past the layer of epithelium into the basement membrane and, as a result, do not invade into the vasculature or nearby lymph nodes [14]. Not all adenomatous polyps or even sessile serrated adenomas progress to invasive adenocarcinoma. In fact, prior meta-analyses, such as the one conducted by Heitman et al., have estimated that less than 5% of all adenomatous polyps without advanced features will progress to become cancerous lesions of the colon [8]. Similarly, high-grade dysplasia is also relatively rare in adenomatous polyps, present in only roughly 5%-7% of such polyps [10].

There are also various benign submucosal polypoid lesions encountered during colonoscopy that do not require endoscopic or surgical intervention. Lymphoid aggregates represent a normal grouping of lymphoid cells, such as histiocytes, lymphocytes, and plasma cells, that do not possess the risk for malignant transformation and thus do not require surveillance [15]. Perineuriomas or fibroblastic polyps are benign tumors of perineural cells that are not cancerous and also do not necessitate surveillance [16]. The most common type of benign submucosal polyp found in the colon is a lipoma, essentially a proliferation of adipose tissue cells. These are classically noted on endoscopy to have a yellow color and soft texture, as exhibited by the “pillow sign” when gentle pressure applied with endoscopic forceps or other probe produces indentation on the polyp [17]. Once again, lipomas do not require further surveillance colonoscopy.

Table 1 summarizes colonic polyp types and their key characteristics.

**Table 1:** Colonic polyp types and key characteristics SSA: sessile serrated adenoma, TSA: traditional serrated adenoma Table credits: John Azizian Sources: [1-17]

Polyp type	Endoscopic appearance	Histologic features	Malignant potential
Hyperplastic polyp	Small, pale, smooth; similar to normal mucosa [1-3]	Non-dysplastic, normal proliferation [1-3]	Very low (especially small, left-sided lesions) [1-3]
Inflammatory polyp	Irregular, regenerating mucosa [4]	Reactive, non-neoplastic tissue [4]	None [4]
SSA	Flat, mucus cap, subtle borders [5,6]	Serrated (sawtooth) architecture [5,6]	Increased [5,6]
TSA	Polypoid, serrated appearance [7]	Ectopic crypts, pseudostratification, villous features [7]	High [7]
Adenomatous polyp - tubular	Pedunculated or sessile [12]	Branching tubular glands [12]	Low-moderate [8-12]
Adenomatous polyp - villous	Sessile, larger [12]	Finger-like villous projections [12]	High [10-14]
Adenomatous polyp - tubulovillous	Mixed morphology [12]	Combination of tubular and villous features [12]	Intermediate-high [10-14]
Advanced adenoma	≥10 mm, often sessile [10-14]	May include villous features or high-grade dysplasia [10-14]	High [10-14]
Lymphoid aggregate	Small, submucosal nodule [15]	Normal lymphoid tissue [15]	None [15]
Perineurioma (fibroblastic polyp)	Small, sessile [16]	Perineural cell proliferation [16]	None [16]
Lipoma	Yellow, soft, “pillow sign” [17]	Adipose tissue [17]	None [17]

Guidelines for colorectal cancer screening and detection of pre-cancerous polyps

Colorectal cancer remains a significant concern today, as according to the American Cancer Society, about 154,000 new cases of colorectal cancer are diagnosed each year in the United States (representing the third highest prevalence among all cancers) [18]. Colorectal cancer is also responsible for roughly 53,000 deaths nationally per year, placing it second-highest among all cancers in the United States [18]. In addition, although the incidence of colorectal cancer steadily decreased in the United States from 2012 to 2021 by 1% per year on average, the incidence in patients younger than 50 increased by an average of 2.4% each year during that same time period [18]. This troubling trend highlights the importance of early detection, removal, and subsequent tracking of pre-cancerous polyps, especially those with advanced features or high-grade dysplasia.

To this end, it is now recommended by the United States Multi-Society Task Force (USMTF) on colorectal cancer to begin screening patients with either colonoscopy or yearly fecal immunohistochemical test (FIT) at age 45 if at average risk [19]. If a patient has a first-degree relative who was diagnosed with colorectal cancer prior to age 60, then screening is recommended to begin at age 40 or 10 years younger than the age at which the said relative was diagnosed with colorectal cancer (whichever age comes first) [19].

It is also widely recommended and accepted in clinical practice that in patients with average risk for colorectal cancer who undergo index screening colonoscopy (assuming bowel prep was adequate for good visualization) that does not detect any polyps or other abnormalities, the next colonoscopy for screening purposes should be repeated in 10 years [19]. This interval tends to be shortened, however, if the family history of colorectal cancer is significant.

In most patients without a history of advanced adenomas, having only a small cumulative lifetime number of adenomatous polyps smaller than 10 mm in size, or having no history of polyps ever detected by colonoscopy, the recommended age for final colonoscopy is 75 [19]. Colonoscopies are not without risk, although these are lower in younger, healthier patients. Procedural risks include infection (low due to utilizing sterile endoscopic equipment); prolonged bleeding after polypectomies, especially if patients are on anticoagulation that cannot be held prior to the procedure; and colonic perforation, which, while present in roughly only 3 in 10,000 cases, can be a life-threatening emergency [20]. Thus, after age 75, there should be shared decision-making between the gastroenterologist and the patient to discuss whether to continue post-polypectomy surveillance or not. This counseling process should consider the patient’s comorbidities and overall life expectancy, and weigh both the procedural and anesthesia risks involved for the patient [20].

Furthermore, even in colonoscopies with high-quality bowel prep allowing excellent visualization, there is still a risk of missed polyps or lesions, and it is the goal of AI technology to help minimize this risk. In a large meta-analysis by Zhao et al. that included 15,000 colonoscopies conducted without AI compiled across 43 studies, researchers estimated the rate of all missed polyps to be approximately 26%, while roughly 9% of advanced adenomas were missed on index colonoscopy [21]. Smaller polyps are more likely to be missed, but any polyp may not be visualized at a given moment due to location within or next to colonic folds. In addition, as expected, the lower the quality of the bowel prep (some patients may necessitate two days of bowel prep for adequate prep), the higher the chances of missing a polyp during colonoscopy [22]. This highlights the importance of counseling the patient in the pre-procedure period to closely follow the instructions for bowel prep and confirming clear yellow stools prior to the colonoscopy, in order to best minimize the risk of missing polyps during the procedure [22].

While adenoma detection and removal during screening or surveillance colonoscopy is associated with improved mortality, differences exist between individual endoscopists, and procedural results may vary. Thus, a target adenoma detection rate was first proposed as a key quality indicator in 2002 and later adopted by the American Society of Gastroenterology (ASGE) and the American College of Gastroenterology (ACG) in 2006 [23,24]. The adenoma detection rate is defined as the proportion of screening colonoscopies that detect at least one histologically confirmed colorectal adenoma or adenocarcinoma. Currently, the benchmark adenoma detection rate for the general population (regardless of gender) at average risk for colorectal cancer has been increased from the previous target of 25% to 35% as of new guidelines in 2024, while gender-specific targets are 30% in men and 20% in women [23,24]. 

In 2017, the USMTF on colorectal cancer had suggested that these benchmarks should be even higher and recommended an adenoma detection rate target greater than 45% for men and greater than 35% for women [25]. However, it was acknowledged that this was a “weak recommendation” based on “very low quality evidence,” partly because it is challenging to establish specific benchmarks for adenoma detection rate in FIT-positive patients who have been screened to undergo a colonoscopy, when the hemoglobin threshold for a positive test varies depending on the specific test kit [25,26]. However, these heightened target adenoma detection rates were not adopted into common practice due to the need for more robust evidence in further research, as well as the aforementioned variance between individual endoscopists.

Recommendations for surveillance colonoscopies after index colonoscopy polyp detection

Once again, USMTF guidelines that are widely accepted in practice by major gastroenterological societies in the United States help advise when a patient’s colonoscopy should be repeated if polyps are detected. With the assumption that a patient with average risk for developing colorectal cancer in their lifetime underwent the colonoscopy with adequate bowel prep and endoscopic visualization, timing for the subsequent surveillance colonoscopy is determined by the size, type, and number of polyps encountered (as summarized in Table 2, but elaborated on in greater detail in the following text).

**Table 2:** Recommended surveillance intervals following colonoscopy and polypectomy, adapted from the 2020 US Multi-Society Task Force on Colorectal Cancer guidelines Intervals assume a high-quality baseline colonoscopy with complete polyp resection and adequate bowel prep. SSL: sessile serrated lesion Table credits: John Azizian Sources: [19,25]

Index colonoscopy finding	Definition	Recommended interval
Normal colonoscopy	No adenomas or serrated lesions ≥10 mm	10 years
1-2 tubular adenomas <10 mm	Low-grade dysplasia	7-10 years
3-4 tubular adenomas <10 mm	All <10 mm	3-5 years
5-10 adenomas <10 mm	Any histology	3 years
≥1 adenoma ≥10 mm	Any histology	3 years
Villous histology	Any size	3 years
High-grade dysplasia	Any size	3 years
>10 adenomas	Complete removal	1 year
SSL <10 mm (no dysplasia)	1-2 lesions	5-10 years
SSL ≥10 mm or dysplasia	Any number	3 years
Traditional serrated adenoma	Any size	3 years
Piecemeal resection ≥20 mm	Uncertain complete excision	6 months

It is important to note that if the bowel prep was inadequate, colonoscopy, regardless of whether polyps were detected, should be repeated within a year [19]. If the prep was fair, and it was deemed by the endoscopist that polyps could be visualized if larger than 5 mm, then repeat colonoscopy, at the provider’s discretion, is usually recommended in 3-5 years, although no clear guidelines exist in this respect. AI technology has its limitations with inadequately prepped colonoscopic examinations, since stool particles might be “flagged” as potential polyps due to their shape and appearance.

The following recommendations outlined in this section are for cases with adequate bowel prep, no significant family history of colorectal cancer, complete resection of any polyps that were encountered, and no prior personal history of having multiple polyps, advanced adenomas, or colorectal cancer diagnosed during colonoscopy.

If 1-2 tubular adenomas (without high-grade dysplasia) smaller than 10 mm in size are encountered during colonoscopy, then repeat surveillance colonoscopy is recommended in 7-10 years [19]. In patients who have 3-4 tubular adenomas (without high-grade dysplasia) smaller than 10 mm in size found during colonoscopy, repeat surveillance colonoscopy is recommended in 3-5 years [19]. For those who undergo a colonoscopy that detects 5-10 tubular adenomas (without high-grade dysplasia) smaller than 10 mm in size, a repeat colonoscopy is recommended in 3 years [19].

If a patient undergoes a colonoscopy that detects one or more adenomatous polyps that are larger than 10 mm in size, then a repeat colonoscopy is suggested in 3 years [19]. In patients in whom a villous adenoma was detected during colonoscopy, it is once again recommended to undergo the next colonoscopy in 3 years [19]. Similarly, for patients who undergo a colonoscopy that detects any polyp with high-grade dysplasia, a repeat colonoscopy is recommended in 3 years [19].

Patients who are found to have more than 10 adenomatous polyps (of any kind, including sessile serrated adenomas and traditional serrated adenomas) during a colonoscopy are advised to have their colonoscopy repeated in 1 year [19].

Since hyperplastic polyps generally have no significant risk for malignant transformation, there are much more lenient parameters involved in their follow-up surveillance colonoscopy recommendations than for adenomatous polyps. The exception to this rule is if the hyperplastic polyp is larger than 10 mm or if there are greater than 20 found during the colonoscopy. For patients who have less than 20 hyperplastic polyps found during colonoscopy (without any other adenomatous polyps found), it is recommended to have the repeat surveillance colonoscopy in 10 years, the same recommended timing as if no polyps were encountered at all [19]. This is the same whether hyperplastic polyps were found in the rectum, sigmoid colon, or even descending colon or cecum [19].

If a patient undergoes a colonoscopy that detects a large hyperplastic polyp greater than 10 mm in size that is completely resected, without any other adenomatous polyps encountered, then the surveillance colonoscopy should be repeated in 3-5 years [19]. Determining whether the repeat examination is in 3 or 5 years depends chiefly on how confident the endoscopist (based on the morphological appearance of the polyp) and pathologist are that the diagnosis of a hyperplastic polyp is correctly favored over a sessile serrated adenoma, as well as the quality of the bowel prep.

Since sessile serrated adenomas have a higher risk for malignant transformation, it is recommended that there be less time between index and surveillance colonoscopies compared to similarly sized adenomatous polyps [19]. Patients who have 1-2 sessile serrated adenomas smaller than 10 mm in size identified and resected on index colonoscopy are recommended to have their colonoscopy repeated in 5-10 years [19]. For those who have 3-4 sessile serrated adenomas detected that are smaller than 10 mm in size, repeat surveillance colonoscopy is recommended to take place in 3-5 years [19]. In patients who are found to have 5-10 sessile serrated adenomas of any size, it is recommended to undergo surveillance colonoscopy in 3 years [19]. Surveillance colonoscopy is repeated in 3 years for patients who undergo colonoscopy and are found to have one sessile serrated adenoma larger than 10 mm in size, similar to the recommendation for other adenomatous polyps larger than 10 mm [19].

For patients who have a sessile serrated adenoma with any degree of dysplasia (even low grade), it is recommended to have a repeat colonoscopy in 3 years [19]. Any patient who is found to have one or more traditional serrated adenomas should undergo surveillance colonoscopy in 3 years, due to the aforementioned heightened risk for malignant transformation in these polyps [19].

Adenomatous polyps of any kind that are 20 mm or larger in size will usually require piecemeal resection during colonoscopy, and for these patients, it is recommended to repeat their colonoscopy in only 6 months [19].

If a patient has had a prior screening colonoscopy during which time some number of adenomatous polyps (also including sessile serrated adenomas and traditional serrated adenomas) were removed and then underwent surveillance colonoscopy for their subsequent procedure, then the recommendation for when to repeat the colonoscopy after this surveillance endoscopic examination should depend on the type and number of polyps that were found during both the last screening colonoscopy and first surveillance colonoscopy [19].

If the initial screening colonoscopy in a patient with average risk for colorectal cancer did not have any advanced lesions and less than 10 adenomas, while the surveillance colonoscopy did not yield any adenomas at all, then the next surveillance colonoscopy should be performed in 10 years [19]. In patients with similar screening colonoscopy findings and only 1-2 small adenomas less than 10 mm in size found during surveillance colonoscopy, a second surveillance colonoscopy is recommended to occur in 7-10 years [19].

If surveillance colonoscopy in these patients yields 3-4 adenomas smaller than 10 mm, then subsequent surveillance should be performed in 3-5 years [19]. If surveillance colonoscopy in these patients yields an advanced adenoma or 5-10 adenomas smaller than 10 mm, then the following surveillance colonoscopy should occur in 3 years [19]. Furthermore, if instead surveillance colonoscopy in this cohort (with the same screening colonoscopy findings) identifies more than 10 total pre-cancerous polyps, regardless of size, then the next surveillance colonoscopy is recommended to take place in 1 year [19].

Finally, in patients who underwent screening colonoscopy that did not detect advanced adenomas and less than 10 total polyps, but who possess a higher risk for developing colorectal cancer (i.e., those with first-degree relatives with colorectal cancer or advanced adenomas prior to age 60), subsequent surveillance colonoscopies tend to take place at least once every 5 years, and even sooner as stated above if advanced adenomas or more than 10 total polyps are present [19].

As expected, if the initial screening colonoscopy did in fact identify an advanced adenoma, then the timing for subsequent surveillance colonoscopies after the first surveillance examination is slightly different. In general, because patients with advanced adenomas identified at any point in their lives still appear to be at higher risk for colorectal cancer, they tend to necessitate shorter baseline surveillance follow-up intervals throughout their lives [19]. If these patients had a first surveillance colonoscopy that did not detect any adenomatous polyps at all, then subsequent surveillance colonoscopy should take place in 5 years [19]. Similarly, if only 1-2 tubular adenomas smaller than 10 mm in size are found during the first surveillance colonoscopy, then the next surveillance colonoscopy should also take place in 5 years [19].

For these patients with a higher than average lifetime risk of having colorectal cancer, who undergo a first surveillance colonoscopy that identifies 3-4 tubular adenomas smaller than 10 mm in size, without the presence of sessile serrated adenomas or traditional serrated adenomas, the next surveillance colonoscopy should be performed in 3-5 years [19]. Generally speaking, if a small sessile serrated adenoma or traditional serrated adenoma is present during the first surveillance colonoscopy, and the total number of adenomatous polyps is less than 10, the subsequent surveillance colonoscopy also tends to occur in 3-5 years [19]. If these patients with a history of advanced adenomas or prior colonoscopy, but not more than 10 polyps, have a surveillance colonoscopy that again identifies an advanced adenoma or 5-10 adenomatous polyps, then the subsequent surveillance colonoscopy should also take place in 3 years [19].

If a patient has more than 10 adenomatous polyps encountered during a single colonoscopy, or even cumulatively throughout their lifetime, some studies suggest evaluating them for a possible hereditary colorectal cancer syndrome [19,27,28]. We will expand further on specific considerations for some of these genetic cancer and polyp-producing syndromes and conditions in later sections.

Recommendations for surveillance colonoscopies after colorectal cancer diagnosis

If a patient has been diagnosed with invasive adenocarcinoma or another colonic malignancy that does not include metastases from other primaries, the algorithm for when to repeat colonoscopies is slightly different. These patients should promptly have surgical resection of the tumor, unless possible to fully resect endoscopically with endoscopic mucosal resection (EMR) and demonstrate clear margins. Oftentimes, the tumor site is marked with endoscopic tattoo ink injection. Oncology should also be consulted, and a multidisciplinary approach should be taken to provide the patient with the best possible care.

In compliance with USMTF guidelines, because patients who have undergone surgical resection of invasive colonic adenocarcinoma have approximately double the risk as the general population to develop new pre-cancerous or cancerous polyps, these individuals should have their first subsequent colonoscopy 1 year after surgical resection [19]. The second such surveillance colonoscopy should take place 3 years after that, and all other following surveillance colonoscopies should occur every 5 years [19].

It is important to note that the USMTF also posits that patients with a personal history of colorectal cancer should not be given the option to perform FIT stool testing in lieu of colonoscopies for surveillance [19]. In general, FIT is a non-invasive screening test to help triage patients toward a colonoscopy; the colonoscopy that follows a positive FIT is thus not purely screening since this has been “flagged,” in a manner of speaking, to be higher risk for a significant polyp or even malignancy by the positive stool test. However, FIT has a false-positive rate of roughly 39%-52%, meaning that these patients did not have adenomatous polyps (advanced or otherwise) or invasive adenocarcinoma found during colonoscopy [29].

Polypectomy guidelines and post-polypectomy complications

When a polyp is encountered during any colonoscopy, unless it is very large or encompasses the entire circumference of the lumen, thus raising concern for invasive adenocarcinoma, complete resection should be attempted. Polyps 2 mm or smaller in size can usually be completely resected with forceps [30]. Polyps that are 3-10 mm in size can usually be resected in one attempt (not piecemeal) with cold snare polypectomy [30]. Cold snare polypectomy has the advantage of carrying a lower risk for healthy mucosal tissue injury around the polyp site, thus minimizing post-polypectomy bleeding risk [31]. Large sessile adenomas often require piecemeal resections with or without the EMR technique [32].

Polyps that are larger than 10 mm may require hot snare electrocautery polypectomy in an attempt to resect all edges of the larger polyp and minimize procedural bleeding, but this carries its own risk of complications, both peri-procedural and post-procedural, that are vital to be aware of. Post-polypectomy bleeding occurs in 0.02%-2% of all polypectomies, with increased risk for polyps larger than 10 mm and an even greater risk for polyps greater than 20 mm in size, pedunculated polyps, and polyps in the right colon [33]. For this reason, it is generally recommended to place clips after performing EMR of polyps larger than 20 mm in an attempt to reduce post-polypectomy bleeding rate, and hemostatic clip placement with or without epinephrine injection is the most common post-polypectomy bleeding intervention [34]. The risk for colonic perforation also increases for resected polyps that are larger than 20 mm and those in the right colon [35]. This could lead to sepsis and hemodynamic instability, along with abdominal pain, with CT of the abdomen showing free air, which often necessitates prompt surgical intervention in the form of primary closure versus bowel resection if the perforation involves a large area of the bowel.

A rarer but still significant complication of hot snare electrocautery polypectomy is post-polypectomy coagulation syndrome, which occurs in roughly 1% of cases utilizing the hot snare technique [36]. This condition arises when electric current extends beyond the mucosa to the layer of the muscularis propria and serosa, which can result in peritonitis [36]. Patients present with symptoms similar to those seen with colonic perforations, and risk is once again increased for polyps in the right colon and those that are larger than 20 mm in size [37]. However, risk for post-polypectomy coagulation syndrome is also increased in patients with hypertension and those with non-polypoid flat adenomatous lesions [37]. Unlike a colonic perforation post-polypectomy, CT imaging does not show free air in the abdomen and instead shows severe mural wall thickening [37]. Surgery is rarely necessary if the patient improves (generally within 2-3 days) with supportive care measures that include only clear liquids or NPO diet, a 7-day antibiotic course, and intravenous fluids for hydration [37].

En bloc resection (removing the entire polyp in addition to healthy surrounding mucosal tissue) is the recommended technique utilized to remove pedunculated polyps that are suspicious for deep submucosal invasion [38]. Non-pedunculated polyps that are suspicious for deep submucosal invasion should be biopsied and tattooed to mark their location, since they will most likely require surgery for complete resection [39]. However, non-pedunculated polyps believed to have more superficial submucosal invasion may be resected endoscopically [39]. Incomplete resection of flat or sessile polyps can increase the risk for future development of colorectal cancer, and studies have shown that the risk for incomplete polyp resection increases with polyp size [40]. For this reason, ensuring that the full margin of the polyp (with some surrounding normal mucosa) is resected and confirming normal margins on pathology are vital in reducing the risk for developing invasive colorectal cancer later in life [40].

If a polyp is confirmed to be invasive carcinoma by pathology and possess any one of the following characteristics: poorly differentiated histology, lymphovascular invasion, tumor budding or islanding throughout other healthy mucosal tissue, submucosal invasion depth greater than 1 mm, or positive margins (cancer present less than 2 mm from resected margin), then the patient should be referred to surgery for possible colectomy [38].

While surgery may often still be necessary for the removal of large polyps (those at least 20 mm in size), many are first attempted to be resected endoscopically in order to avoid subjecting a patient to the risks involved with surgery. These polyps must be accessible endoscopically, have low invasive cancer risk (i.e., no deep submucosal layer invasion of 1 mm or greater into the SM2 layer), and not carry an increased risk for post-polypectomy perforation or bleeding [38]. Pedunculated polyps with a normal-appearing stalk can be resected by polypectomy [38]. As mentioned previously, snare polypectomy without submucosal injection for polyp lifting is generally sufficient to remove polyps up to 10 mm in size.

EMR, however, for larger polyps, involves injection of saline or other solution into the submucosa to raise the mucosal layer and ensure that piecemeal polypectomies (recommended for polyps that are 20 mm or larger to decrease risk of perforation) utilizing a snare get adequate margins, which can also be helpful in removing laterally spreading tumors [41]. The underwater form of this technique avoids the use of submucosal injection and uses water’s ability to “float” the polyp and separate mucosal/submucosal layers [42]. Endoscopic submucosal dissection (ESD) is a more complex procedure requiring specialized advanced training that uses a dissection knife to allow for en bloc removal of a large polyp [43].

Failure of a polyp to lift when injected with solution may indicate a more likely colorectal cancer that has invaded the submucosa [44]. However, a non-lifting sign has a relatively high false-positive rate (~30% in some studies), and it is believed that endoscopic ultrasound has more sensitivity for identifying a submucosal lesion [45].

Polyps that are one-third the size of the full luminal circumference, cross two haustral folds, or are located at the base of the appendix should undergo EMR or ESD, oftentimes with an advanced interventional endoscopist [46]. Endoscopic polyp removal is contraindicated in patients who have multiple large right colon polyps (due to increased risk for perforation with multiple endoscopic procedures needed for complete removal), an inability to follow up for repeated surveillance and re-treatment colonoscopies post-polypectomy, or an uncorrectable bleeding disorder [47].

After a large polyp is resected, high-definition white light endoscopy or narrow band imaging can be used to check for remnant adenomatous tissue. It is important to minimize multiple resections of the same polyp site across different endoscopic sessions because scar tissue can make further evaluation and treatment of the site more challenging. Thermal ablation with argon plasma coagulation (APC) of the resected margins can help reduce adenomatous recurrence related to remnant pre-cancerous tissue post-polypectomy [48]. The success rate for EMR is high at about 95%, while the rate of recurrent adenomas post-EMR ranges from 5% to 30% [41,42].

It is generally accepted good practice to tattoo near the site of large polyp resection, 3 cm away from the site, so as not to confuse the margins of the site later, in order to mark the site for subsequent colonoscopies or, if needed, surgery [49]. It is also helpful to inject the stalk of pedunculated polyps with epinephrine (if the stalk is larger than 10 mm) to minimize post-polypectomy bleeding [50].

Serrated polyposis syndrome

Diagnosis of serrated polyposis syndrome (SPS) is made if a patient has either more than 20 serrated polyps throughout the colon (although at least 5 must be proximal to the colon) or at least 5 serrated polyps proximal to the rectum that are all at least 5 mm in size and at least 2 polyps that are 10 mm in size or larger [51]. This syndrome is associated with increased colorectal cancer risk, believed to be due to the high rate of dysplasia and high incidence of advanced adenomas among serrated adenomas [52]. Surveillance colonoscopy for patients with this rare condition is usually repeated every 1-3 years [52]. Patients with first-degree relatives with SPS are recommended to begin screening at age 40 or 10 years before the age of diagnosis of the said first-degree relative (whichever age comes first) and have screening colonoscopies every 5 years if no polyps are found [52]. Patients may require colectomy if a diagnosis of colorectal cancer is confirmed, there are multiple polyps with high-grade dysplasia or that are larger than 6 mm in size, or if there is an increasing number of polyps detected on subsequent surveillance colonoscopies [27].

Peutz-Jeghers syndrome (PJS)

Peutz-Jeghers syndrome (PJS) is a rare autosomal dominant condition that increases the risk of having gastrointestinal and other cancers, as well as hamartomatous colonic polyps [53]. It is caused by a mutation in the *STK11* tumor suppressor gene that codes for a serine threonine kinase [54]. Most patients will have polyps found by age 30, while 10%-20% of mutations are believed to be de novo without any family history [54].

Most polyps in PJS occur in the small intestine (specifically the jejunum), but colonic polyps are present in 50%-64% of these patients [55]. Hamartomatous polyps can also arise in other organs, including the kidneys, bladder, lungs, and head/neck region [55]. Intussusception due to a large polyp in the small bowel is also more likely to occur at some point during these patients’ lifetime [56].

Hamartomatous polyps can vary in shape, number, and size (as small as 1 mm to as large as greater than 5 cm) and do not have any specific discerning morphological qualities until evaluated microscopically, where smooth muscle proliferating into the lamina propria in a tree-like fashion is classically observed [57]. PJS carries an estimated lifetime risk for gastrointestinal cancer of 38%-66%, with 39% of patients developing colorectal cancer, and a median age of cancer diagnosis of 42 [57]. It is key for even gastroenterologists to counsel the patient on the importance of screening for primary malignancies outside the gastrointestinal tract and refer him or her to other specialists as needed. Other common locations for malignancies in this patient population include the pancreas, ovaries, endometrium, testes, cervix, and breast, among others [58].

Individuals should be tested for *STK11* mutation if they have two or more hamartomatous polyps seen endoscopically, regardless of family history, any hamartomatous polyp encountered endoscopically if a first-degree relative has PJS, or a characteristic mucocutaneous hyperpigmented rash of the mouth, lips, nose, eyes, fingers, or genitalia [57].

USMTF guidelines currently recommend that these patients undergo initial screening for gastrointestinal malignancy either at the age of diagnosis or as early as 8-10 years of age (if the diagnosis is made earlier with positive family history for the syndrome leading to confirmatory genetic testing), with esophagogastroduodenoscopy (EGD) to evaluate the stomach and duodenum, video capsule enteroscopy or MR enterography to evaluate the small bowel, and colonoscopy [59]. If any polyps are detected on initial screening, then all three of the aforementioned screening endoscopic or imaging examinations should be repeated every 2-3 years [59]. If no polyps are found on initial screening before age 18, the subsequent surveillance examinations can be held until age 18 and then once again repeated every 2-3 years [59].

Familial adenomatous polyposis (FAP) syndrome

Familial adenomatous polyposis (FAP) is another rare autosomal dominant condition that results from a mutation in the adenomatous polyposis coli (*APC*) gene and is classically associated with the presence of more than 100 (and often into the 1000’s) colorectal adenomatous polyps [60]. The presence of such a large number of pre-cancerous polyps all throughout the colon that vary in size conveys a 100% chance of developing colorectal cancer in the future, in addition to other malignancies found outside the colon [60].

Early identification of individuals with this genetic disorder is once again of the utmost importance. Patients who have first-degree relatives diagnosed with FAP should have genetic testing for the *APC* gene mutation [27]. In addition, as stated in a previous section, *APC* gene testing should be performed on individuals who are found to have more than 10 adenomatous polyps during one colonoscopy [27]. Genetic testing should also be performed on individuals who are found to have adenomatous polyps in combination with extraintestinal lesions characteristic of FAP, including osteomas, epidermal cysts, retinal pigment epithelium hamartomas associated with FAP (RPEH-FAP), duodenal or ampullary adenomas, desmoid tumors, and papillary thyroid cancer [27].

Once a diagnosis of FAP is made, the patient should be prepared for eventual colectomy at some point in the future. If early diagnosis based on family history and genetic testing is made, the first colonoscopy should occur as early as 10-15 years of age, with yearly surveillance colonoscopies performed to ensure no progression to colorectal cancer prior to colectomy [61]. During these surveillance colonoscopies, the estimated number of polyps should be documented, while multiple polyps should ideally be resected for confirmatory pathologic evaluation [61]. However, if there are too many polyps to feasibly remove during the colonoscopy, then the topic of colectomy should be discussed expediently [61].

For those patients who have been diagnosed with FAP (due to family history and *APC* mutation), but no polyps are identified on screening colonoscopy by the age of 15, the time between screening colonoscopies can be lengthened from the standard 1-year interval to every 2 years instead, although lifelong screening colonoscopies should still be performed [61]. In those who are diagnosed with FAP later in life (after age 15), either through colonoscopic evidence of FAP plus subsequent *APC* gene mutation or based on family history plus *APC* gene mutation, surveillance colonoscopies again tend to occur every 1 or 2 years, respectively, until colectomy is performed [61].

Colectomy should be performed in patients with FAP if a diagnosis of colorectal adenocarcinoma is made, polyposis burden is so high that it causes rectal bleeding, polyps have high-grade dysplasia or are larger than 6 mm in size, or polyps are too numerous to be effectively resected endoscopically [61]. The specific extent of colectomy is determined by the distribution of polyps, as well as how many are present in the rectum, which could guide the need for proctectomy in addition to colectomy; the specific type of *APC* gene mutation can also be helpful in guiding the extent of colectomy that is best for the patient [27,61]. Even if patients have undergone a total proctocolectomy, there remains a risk for adenocarcinoma formation in the ileal pouch (as well as the rectum, if it was not removed during the colectomy), and thus, patients with FAP post-colectomy should have surveillance colonoscopies every 6-12 months [62].

Furthermore, patients with FAP should undergo screening EGDs with a side-viewing scope to also evaluate for duodenal adenomas either at age 20-25 or at the age of detection of colonic polyposis (whichever comes first) [63]. If no duodenal polyps are detected, regardless of the number of non-dysplastic gastric polyps, EGD should be repeated in 4 years [27]. Fundic gland polyps are most common in this patient population and should be resected endoscopically if large/irregular, while smaller polyps can be biopsied to assess for dysplasia [64]. The presence of high-grade dysplasia in gastric polyps should prompt evaluation for possible gastrectomy [64].

On the other hand, the presence of duodenal polyps (which should be completely resected endoscopically) will shorten the time interval between repeat surveillance EGDs based on the severity of polyposis [27,65]. An irregular-appearing ampulla or peri-ampullary lesions should be biopsied [27,65].

Lynch syndrome

Patients with Lynch syndrome carry an increased risk of colorectal as well as gastric, small bowel, ovarian, endometrial, prostate, brain, skin, and pancreaticobiliary cancers [66]. Similar to patients with PJS, gastroenterologists should be wary of their patients with Lynch syndrome possibly having a cancer outside of the gastrointestinal tract and should recommend proper screening and follow-up with other providers or specialists for all organ systems that may be affected. Lynch syndrome is an autosomal dominant disorder caused by a mutation in a DNA mismatch repair gene, including *MLH1*, *MSH2*, *MSH6*, *PMS2*, or *EPCAM* [66,67]. For this reason, genetic testing should be performed in individuals with a suspicion for Lynch syndrome based on a significant family history of the condition.

The optimal colonoscopy screening interval for patients with Lynch syndrome is not clearly defined but is tied to the type of mismatch repair gene mutation each patient has. *MLH1* and *MSH2* gene mutations would suggest that a patient should have a screening colonoscopy every 1-2 years starting at age 20-25 or 2-5 years prior to the earliest age of colorectal cancer diagnosis in a first-degree family member (whichever comes first) [68,69]. Those with mutations in *MSH6 *or *PMS2* should begin screening colonoscopies at age 30-35 and repeat colonoscopy every 3 years as long as there is no family history of early-onset colorectal cancer [68,69].

EGD to screen for gastric cancer is performed first at age 30-35 in patients with Lynch syndrome and repeated every 2-4 years [27,70]. More frequent screening EGDs are performed for patients with Lynch syndrome who have a personal increased risk for gastric cancer, including advanced atrophic gastritis, extensive intestinal metaplasia, autoimmune gastritis, or family history significant for gastric cancer before the age of 50 or diagnoses of multiple gastric cancers in first-degree relatives [27,70]. Moreover, individuals with Lynch syndrome who are of East Asian descent are also thought to have an elevated risk for developing gastric cancer and thus tend to get screening EGDs performed more frequently [27,70].

Small bowel malignancy is less common in Lynch syndrome, but if present, it tends to occur more in the duodenum or terminal ileum [68]. For this reason, it is a good practice to carefully inspect both these anatomical regions during screening EGD and colonoscopy.

If a cancerous polyp or a pre-cancerous polyp is encountered on screening colonoscopy or EGD, referral to surgery should be made if the polyp is not resectable endoscopically [27,71]. Those found to have colon cancer requiring colectomy are recommended to have total colectomy with ileorectal anastomosis [68,71]. Patients who decline total colectomy can have a segmental colectomy but then must undergo surveillance colonoscopies consistent with the guidelines post-cancer diagnosis discussed in prior sections, and if a second colon cancer is discovered upon surveillance, then undergo total colectomy [71]. If rectal cancer is detected, patients should undergo total proctocolectomy with ileal pouch anal anastomosis [72,73]. Neoadjuvant chemotherapy may follow colectomy or proctocolectomy, and involving oncology in these cases is encouraged.

Primary sclerosing cholangitis (PSC)

Primary sclerosing cholangitis (PSC) is a fibrotic, stricturing disease of the medium and large biliary ducts that appears to be linked to ulcerative colitis (UC) and thus carries an increased risk for colorectal cancer [74]. If a patient with PSC (who otherwise does not have other reasons to have elevated colorectal cancer risk) does not also have a diagnosis of UC, screening colonoscopies are recommended to take place every 3-5 years [75]. In patients with PSC with a concurrent diagnosis of UC, it is recommended that screening colonoscopies be performed annually since the combination of PSC and UC significantly increases the risk of developing colorectal cancer further [75].

Table 3 summarizes hereditary polyposis syndrome characteristics, malignancy risks, and screening recommendations.

**Table 3:** Summary of hereditary polyposis syndrome characteristics, malignancy risks, and screening recommendations SPS: serrated polyposis syndrome, PJS: Peutz-Jeghers syndrome, EGD: esophagogastroduodenoscopy, FAP: familial adenomatous polyposis, PSC: primary sclerosing cholangitis Table credits: John Azizian Sources: [27,51-62,66-75]

Syndrome	Genetics/etiology	Key features	Cancer risk	Screening and surveillance
SPS	No well-defined genetic mutation [51]	Numerous serrated polyps throughout colon [51]	Increased risk of colorectal cancer [52]	Colonoscopy every 1-3 years, earlier screening for first-degree relatives [52]
PJS	Autosomal dominant, *STK11* mutation [53-54]	Hamartomatous polyps, mucocutaneous pigmentation, small bowel predominance [53-54]	High risk of gastrointestinal and extraintestinal cancers [55-58]	Early and routine multimodal screening, including EGD, colonoscopy, and small bowel imaging beginning in childhood or adolescence [59]
FAP	Autosomal dominant, *APC* mutation [60]	Hundreds to thousands of adenomatous polyps, extracolonic manifestations [61]	Near-certain risk of colorectal cancer without treatment [60-62]	Early screening starting in adolescence with frequent colonoscopy and consideration of prophylactic colectomy [27,62]
Lynch syndrome	Autosomal dominant, DNA mismatch repair gene mutations including *MLH1*, *MSH2*, *MSH6*, *PMS2*, and *EPCAM* [66,67]	Few polyps but rapid progression to malignancy, associated with extracolonic cancers [66,67]	High risk of colorectal and multiple extracolonic cancers [66-68]	Regular colonoscopy beginning in early adulthood, interval based on mutation type, upper gastrointestinal surveillance as indicated [27,68-73]
PSC	Chronic inflammatory biliary disease, associated with inflammatory bowel disease [74,75]	Biliary stricturing disease, often coexists with ulcerative colitis [74,75]	Increased colorectal cancer risk, especially with concurrent ulcerative colitis [74,75]	Colonoscopy every 3-5 years, more frequent if associated with ulcerative colitis [74,75]

The role of AI in polyp detection, screening, and surveillance

As important as early polyp detection and resection are for the goal of colorectal cancer screening and possibly prevention, as we have discussed previously, human errors do occur. Sometimes, polyps, even large ones, can be missed endoscopically by the human eye. The evolving role of AI in colorectal cancer, and even gastric cancer screening and surveillance, aims to minimize the number of missed adenomas, polyps with dysplasia, other pre-cancers, and even malignant polyps during endoscopic procedures. In this sphere, we have seen various advances and new technologies that can potentially improve both endoscopists’ success rate and patient outcomes.

Computer science and programming enable AI software to be built using “deep learning tools” to review vast annotated databases of imaging of known adenomatous and malignant polyps so that these deep neural networks have a reference point to identify new polyps endoscopically if encountered on examination [76,77]. It is also postulated that the more an AI software is used for this purpose in polyp detection, the more reference points it gains and the “smarter,” less error-prone it can become [76,77]. In addition, imaging enhancements using AI can help improve the quality of visuals available to the endoscopists during the procedure [76,77]. This can aid in proceduralists identifying less obvious polyps, such as those that are flat, serrated adenomas (especially with a fecal cap), or even if bowel prep is suboptimal and stool remnants interfere partially with visualization. Moreover, AI can be invaluable in improving surveillance colonoscopies for patients with inflammatory bowel disease (who, as we have mentioned, carry an elevated risk for colorectal cancer), as tissue with dysplasia that is at higher risk for potentially developing into cancer in the future can be better identified [76,77].

In a landmark prospective randomized control study published in 2019 by Wang et al., it was shown that AI real-time polyp detection software increased polyp and adenoma detection rate during colonoscopy in a statistically significant manner [78]. In this study of over 1,000 patients and a roughly equal control group (standard endoscopy without AI deep learning tools) and study group (colonoscopy with AI deep learning tools), it was found that the adenoma detection rate in the study group with AI was 29% compared to 20% in the control group [78]. Similarly, the mean number of adenomas detected per person in the AI group was higher (0.53) than for the control group (0.31) [78]. The findings of this study have been essentially reproduced and verified by subsequent large studies and meta-analyses, including a 2023 meta-analysis with over 6,000 patients by Huang et al., which similarly found that the adenoma detection rate was higher in colonoscopies utilizing AI computer-aided detection technology than standard colonoscopies without AI [79].

AI software is also improving in differentiating adenomatous, pre-cancerous polyps from hyperplastic polyps, inflammatory polyps, or lipomas, which do not possess the ability to undergo malignant transformation [76,77]. This can greatly reduce unnecessary polypectomies and, as a result, potential complications for the patient during the procedure. Some technologies are even being developed to help determine the depth of submucosal or mucosal invasion of polyps, thus potentially helping to guide the endoscopist’s decision on how to best remove the polyp, whether it is a simple snare polypectomy or if it would require EMR or ESD [76,77]. In this same vein, AI can also be utilized to more accurately identify the precise margins of polyps and, after polypectomy, can “flag” residual polyps that may be left on the edges of the resection site to be completely removed [76,77].

Some critics of AI in the setting of colonoscopies argue that the detection of small adenomatous polyps that may have otherwise been missed and most likely would have had a low risk of transforming into malignancy would actually increase unnecessary polypectomies that do not have a practical benefit, which in turn increases healthcare costs [80]. However, these small polyps would likely grow in size by the time of subsequent screening or surveillance colonoscopy and thus have to be removed nevertheless at that time. Critics also argue that heightened detection of small polyps that some may deem “insignificant” even if they are adenomatous unnecessarily shortens colonoscopy surveillance intervals, therefore exposing patients to unnecessary procedural risk and once again increasing healthcare expenses [80].

Yet, increasing the adenoma detection rate using AI can actually reduce healthcare costs by identifying (or potentially preventing) colorectal cancer earlier than without AI, thereby reducing expenses associated with cancer management, such as chemotherapy and surgery [80,81]. In a recent modeling microsimulation study by Areia et al., it was estimated that colonoscopies utilizing AI for polyp detection in the United States could prevent an additional 7,194 colorectal cancer cases and 2,089 deaths from colon cancer, and save up to $290 million in healthcare costs each year [82]. However, it must be acknowledged that the findings of this simulation may not be applicable to real-world cases in practice.

AI still has limitations in detecting polyps that are missed due to “exposure error,” meaning those that are not visualized due to either being covered by mucosa (a fold of tissue in the colon creating a blind spot, for example) or completely covered in stool when prep is suboptimal [80,81]. Technology to possibly see through such barriers is still being developed and could further improve the utility of AI in endoscopic examinations. Current guidelines continue to recommend proper withdrawal technique to reduce polyps missed due to exposure error, which includes fold examination, adequate colonic distension/insufflation, mucosal cleaning, and appropriate time spent during withdrawal of the colonoscope [80].

Unfortunately, adoption of AI into clinical practice has been slower than expected. It is believed that proceduralist frustration with false-positive polyps detected by AI and issues with appropriate reimbursement from insurance companies for endoscopic procedures performed with AI as compared to without have contributed to AI being integrated less than expected into clinical practice [80]. It has also been suggested by some that perhaps particular endoscopists do not believe there is substantial enough financial gain in performing more surveillance colonoscopies than prior (due to the higher adenoma detection rate associated with using AI during colonoscopies) and are thus reluctant to adopt AI into their daily practice [80].

AI is continuing to grow, evolve, and develop further; it is by no means a tool that has reached its full potential. As its capabilities for deep learning improve, so could its ability to aid in early pre-cancerous polyp detection that may lead to saving numerous lives that could have potentially been otherwise lost due to colorectal cancer. Still, at this time, not all AI detection of polyps is foolproof, and research efforts to continue to optimize the function of AI in this sphere continue. Sometimes, stool, food, irregular colonic folds, and edematous changes in congested enteropathy could all be “flagged” by AI technology during the endoscopic procedure, resulting in the aforementioned false positives [80,81]. The physician must evaluate what is being conveyed to him or her in this instance by the AI technology and accordingly make a determination regarding whether the highlighted lesion/polyp/area is truly one that needs to be resected. It is important for physicians to be critical and skeptical of new technological advancements and innovations, but also simultaneously recognize the benefits their utilization might offer to both their patients and the field of medicine as a whole.

## Conclusions

Colorectal cancer screening relies on effective detection, removal, and surveillance of pre-cancerous lesions. Colonoscopy remains central to this process, with management strategies tailored to individual risk factors and polyp characteristics. Appropriate identification and treatment of lesions, along with consideration of hereditary risk, are essential components of prevention and long-term care.

Artificial intelligence is emerging as a valuable adjunct in colonoscopy, with the potential to enhance lesion detection and improve overall procedural quality. As these technologies continue to evolve and become more integrated into clinical practice, they may support more consistent and accurate identification of clinically significant findings. Continued refinement and adoption of AI-assisted tools may contribute to improved outcomes and advance the effectiveness of colorectal cancer prevention strategies. However, since AI remains a relatively new and rapidly developing resource, limitations still exist in AI-assisted colonoscopy, which illustrates the need for further scientific scrutiny in the form of both retrospective and prospective clinical research studies before widespread implementation in practice nationwide.

## References

[1] Weston AP, Campbell DR (1995). Diminutive colonic polyps: histopathology, spatial distribution, concomitant significant lesions, and treatment complications. Am J Gastroenterol.

[2] Laiyemo AO, Murphy G, Sansbury LB (2009). Hyperplastic polyps and the risk of adenoma recurrence in the polyp prevention trial. Clin Gastroenterol Hepatol.

[3] Dave S, Hui S, Kroenke K, Imperiale TF (2003). Is the distal hyperplastic polyp a marker for proximal neoplasia?. J Gen Intern Med.

[4] Mahmoud R, Shah SC, Ten Hove JR (2019). No association between pseudopolyps and colorectal neoplasia in patients with inflammatory bowel diseases. Gastroenterology.

[5] Higuchi T, Sugihara K, Jass JR (2005). Demographic and pathological characteristics of serrated polyps of colorectum. Histopathology.

[6] Guarinos C, Sánchez-Fortún C, Rodríguez-Soler M (2013). Clinical subtypes and molecular characteristics of serrated polyposis syndrome. Clin Gastroenterol Hepatol.

[7] Jass JR (2007). Classification of colorectal cancer based on correlation of clinical, morphological and molecular features. Histopathology.

[8] Heitman SJ, Ronksley PE, Hilsden RJ, Manns BJ, Rostom A, Hemmelgarn BR (2009). Prevalence of adenomas and colorectal cancer in average risk individuals: a systematic review and meta-analysis. Clin Gastroenterol Hepatol.

[9] Ben Q, An W, Jiang Y (2012). Body mass index increases risk for colorectal adenomas based on meta-analysis. Gastroenterology.

[10] Butterly LF, Chase MP, Pohl H, Fiarman GS (2006). Prevalence of clinically important histology in small adenomas. Clin Gastroenterol Hepatol.

[11] Lieberman D, Moravec M, Holub J, Michaels L, Eisen G (2008). Polyp size and advanced histology in patients undergoing colonoscopy screening: implications for CT colonography. Gastroenterology.

[12] Costantini M, Sciallero S, Giannini A (2003). Interobserver agreement in the histologic diagnosis of colorectal polyps. the experience of the multicenter adenoma colorectal study (SMAC). J Clin Epidemiol.

[13] Wieszczy P, Kaminski MF, Franczyk R (2020). Colorectal cancer incidence and mortality after removal of adenomas during screening colonoscopies. Gastroenterology.

[14] Komuta K, Batts K, Jessurun J, Snover D, Garcia-Aguilar J, Rothenberger D, Madoff R (2004). Interobserver variability in the pathological assessment of malignant colorectal polyps. Br J Surg.

[15] Koboziev I, Karlsson F, Grisham MB (2010). Gut-associated lymphoid tissue, T cell trafficking, and chronic intestinal inflammation. Ann N Y Acad Sci.

[16] Groisman GM, Polak-Charcon S (2008). Fibroblastic polyp of the colon and colonic perineurioma: 2 names for a single entity?. Am J Surg Pathol.

[17] Erginoz E, Uludag SS, Cavus GH, Zengin K, Ozcelik MF (2022). Clinicopathological features and management of colonic lipomas: case reports. Medicine (Baltimore).

[18] (2025). American Cancer Society: Key statistics for colorectal cancer. Last revised.

[19] Gupta S, Lieberman D, Anderson JC (2020). Recommendations for follow-up after colonoscopy and polypectomy: a consensus update by the US Multi-Society Task Force on Colorectal Cancer. Gastroenterology.

[20] Early DS, Ben-Menachem T, Decker GA (2012). Appropriate use of GI endoscopy. Gastrointest Endosc.

[21] Zhao S, Wang S, Pan P (2019). Magnitude, risk factors, and factors associated with adenoma miss rate of tandem colonoscopy: a systematic review and meta-analysis. Gastroenterology.

[22] Chokshi RV, Hovis CE, Hollander T, Early DS, Wang JS (2012). Prevalence of missed adenomas in patients with inadequate bowel preparation on screening colonoscopy. Gastrointest Endosc.

[23] Corley DA, Jensen CD, Marks AR (2014). Adenoma detection rate and risk of colorectal cancer and death. N Engl J Med.

[24] Rex DK, Petrini JL, Baron TH (2006). Quality indicators for colonoscopy. Gastrointest Endosc.

[25] Robertson DJ, Lee JK, Boland CR (2017). Recommendations on fecal immunochemical testing to screen for colorectal neoplasia: a consensus statement by the US Multi-Society Task Force on Colorectal Cancer. Am J Gastroenterol.

[26] Jensen CD, Corley DA, Quinn VP (2016). Fecal immunochemical test program performance over 4 rounds of annual screening: a retrospective cohort study. Ann Intern Med.

[27] Syngal S, Brand RE, Church JM, Giardiello FM, Hampel HL, Burt RW (2015). ACG clinical guideline: genetic testing and management of hereditary gastrointestinal cancer syndromes. Am J Gastroenterol.

[28] Brandaleone L, Dal Buono A, Gabbiadini R (2024). Hereditary colorectal cancer syndromes and inflammatory bowel diseases: risk management and surveillance strategies. Cancers (Basel).

[29] Kim NH, Park JH, Park DI, Sohn CI, Choi K, Jung YS (2017). Risk factors for false fecal immunochemical test results in colorectal cancer screening. J Clin Gastroenterol.

[30] Kaltenbach T, Anderson JC, Burke CA (2020). Endoscopic removal of colorectal lesions-recommendations by the US Multi-Society Task Force on Colorectal Cancer. Gastrointest Endosc.

[31] Augusto Barros R, Monteverde MJ, Federico Barros R, De Elizalde S, Barros AS (2014). Safety and efficacy of cold snare resection of non-polypoid colorectal lesions (0-IIa and 0-IIb). Acta Gastroenterol Latinoam.

[32] van Hattem WA, Shahidi N, Vosko S (2021). Piecemeal cold snare polypectomy versus conventional endoscopic mucosal resection for large sessile serrated lesions: a retrospective comparison across two successive periods. Gut.

[33] Zhang X, Jiang X, Shi L (2024). Risk factors for delayed colorectal postpolypectomy bleeding: a meta-analysis. BMC Gastroenterol.

[34] Gutta A, Gromski MA (2020). Endoscopic management of post-polypectomy bleeding. Clin Endosc.

[35] Htet H, Longcroft-Wheaton G (2024). Prevention of delayed bleeding after resection of large colonic polyps. Best Pract Res Clin Gastroenterol.

[36] Hirasawa K, Sato C, Makazu M, Kaneko H, Kobayashi R, Kokawa A, Maeda S (2015). Coagulation syndrome: delayed perforation after colorectal endoscopic treatments. World J Gastrointest Endosc.

[37] Kim HW (2014). What is different between postpolypectomy fever and postpolypectomy coagulation syndrome?. Clin Endosc.

[38] Shaukat A, Kaltenbach T, Dominitz JA (2020). Endoscopic recognition and management strategies for malignant colorectal polyps: recommendations of the US Multi-Society Task Force on Colorectal Cancer. Am J Gastroenterol.

[39] Hayashi N, Tanaka S, Hewett DG (2013). Endoscopic prediction of deep submucosal invasive carcinoma: validation of the Narrow-band Imaging International Colorectal Endoscopic (NICE) classification. Gastrointest Endosc.

[40] Ono S, Fujishiro M, Goto O, Kodashima S, Omata M (2009). Endoscopic submucosal dissection for colonic laterally spreading tumors is difficult after target tattooing. Gastrointest Endosc.

[41] Belderbos TD, Leenders M, Moons LM, Siersema PD (2014). Local recurrence after endoscopic mucosal resection of nonpedunculated colorectal lesions: systematic review and meta-analysis. Endoscopy.

[42] Binmoeller KF, Weilert F, Shah J, Bhat Y, Kane S (2012). "Underwater" EMR without submucosal injection for large sessile colorectal polyps (with video). Gastrointest Endosc.

[43] Fujishiro M, Yahagi N, Nakamura M (2006). Successful outcomes of a novel endoscopic treatment for GI tumors: endoscopic submucosal dissection with a mixture of high-molecular-weight hyaluronic acid, glycerin, and sugar. Gastrointest Endosc.

[44] Kim BC, Chang HJ, Han KS (2011). Clinicopathological differences of laterally spreading tumors of the colorectum according to gross appearance. Endoscopy.

[45] Han KS, Sohn DK, Choi DH (2008). Prolongation of the period between biopsy and EMR can influence the nonlifting sign in endoscopically resectable colorectal cancers. Gastrointest Endosc.

[46] Waye JD (2005). Advanced polypectomy. Gastrointest Endosc Clin N Am.

[47] Buddingh KT, Herngreen T, Haringsma J, van der Zwet WC, Vleggaar FP, Breumelhof R, Ter Borg F (2011). Location in the right hemi-colon is an independent risk factor for delayed post-polypectomy hemorrhage: a multi-center case-control study. Am J Gastroenterol.

[48] Klein A, Tate DJ, Jayasekeran V (2019). Thermal ablation of mucosal defect margins reduces adenoma recurrence after colonic endoscopic mucosal resection. Gastroenterology.

[49] Kim HG, Thosani N, Banerjee S, Chen A, Friedland S (2015). Effect of prior biopsy sampling, tattoo placement, and snare sampling on endoscopic resection of large nonpedunculated colorectal lesions. Gastrointest Endosc.

[50] Di Giorgio P, De Luca L, Calcagno G, Rivellini G, Mandato M, De Luca B (2004). Detachable snare versus epinephrine injection in the prevention of postpolypectomy bleeding: a randomized and controlled study. Endoscopy.

[51] IJspeert JE, Rana SA, Atkinson NS (2017). Clinical risk factors of colorectal cancer in patients with serrated polyposis syndrome: a multicentre cohort analysis. Gut.

[52] Edelstein DL, Axilbund JE, Hylind LM, Romans K, Griffin CA, Cruz-Correa M, Giardiello FM (2013). Serrated polyposis: rapid and relentless development of colorectal neoplasia. Gut.

[53] Lindor NM, McMaster ML, Lindor CJ, Greene MH (2008). Concise handbook of familial cancer susceptibility syndromes. J Natl Cancer Inst Monogr.

[54] Lim W, Hearle N, Shah B (2003). Further observations on LKB1/STK11 status and cancer risk in Peutz-Jeghers syndrome. Br J Cancer.

[55] van Lier MG, Westerman AM, Wagner A (2011). High cancer risk and increased mortality in patients with Peutz-Jeghers syndrome. Gut.

[56] van Lier MG, Mathus-Vliegen EM, Wagner A, van Leerdam ME, Kuipers EJ (2011). High cumulative risk of intussusception in patients with Peutz-Jeghers syndrome: time to update surveillance guidelines?. Am J Gastroenterol.

[57] Beggs AD, Latchford AR, Vasen HF (2010). Peutz-Jeghers syndrome: a systematic review and recommendations for management. Gut.

[58] Boardman LA, Thibodeau SN, Schaid DJ (1998). Increased risk for cancer in patients with the Peutz-Jeghers syndrome. Ann Intern Med.

[59] Boland CR, Idos GE, Durno C (2022). Diagnosis and management of cancer risk in the gastrointestinal hamartomatous polyposis syndromes : recommendations from the US Multi-Society Task Force on Colorectal Cancer. Gastroenterology.

[60] Vasen HF, Tomlinson I, Castells A (2015). Clinical management of hereditary colorectal cancer syndromes. Nat Rev Gastroenterol Hepatol.

[61] Vasen HF, Möslein G, Alonso A (2008). Guidelines for the clinical management of familial adenomatous polyposis (FAP). Gut.

[62] Parc YR, Olschwang S, Desaint B, Schmitt G, Parc RG, Tiret E (2001). Familial adenomatous polyposis: prevalence of adenomas in the ileal pouch after restorative proctocolectomy. Ann Surg.

[63] Kallenberg FG, Bastiaansen BA, Dekker E (2017). Cap-assisted forward-viewing endoscopy to visualize the ampulla of Vater and the duodenum in patients with familial adenomatous polyposis. Endoscopy.

[64] Rudloff U (2018). Gastric adenocarcinoma and proximal polyposis of the stomach: diagnosis and clinical perspectives. Clin Exp Gastroenterol.

[65] Offerhaus GJ, Giardiello FM, Krush AJ (1992). The risk of upper gastrointestinal cancer in familial adenomatous polyposis. Gastroenterology.

[66] Peltomäki P, Nyström M, Mecklin JP, Seppälä TT (2023). Lynch syndrome genetics and clinical implications. Gastroenterology.

[67] Møller P, Seppälä T, Bernstein I (2017). Incidence of and survival after subsequent cancers in carriers of pathogenic MMR variants with previous cancer: a report from the prospective Lynch syndrome database. Gut.

[68] Giardiello FM, Allen JI, Axilbund JE (2014). Guidelines on genetic evaluation and management of Lynch syndrome: a consensus statement by the US Multi-Society Task Force on Colorectal Cancer. Am J Gastroenterol.

[69] Stjepanovic N, Moreira L, Carneiro F, Balaguer F, Cervantes A, Balmaña J, Martinelli E (2019). Hereditary gastrointestinal cancers: ESMO clinical practice guidelines for diagnosis, treatment and follow-up†. Ann Oncol.

[70] Kim J, Braun D, Ukaegbu C (2020). Clinical factors associated with gastric cancer in individuals with Lynch syndrome. Clin Gastroenterol Hepatol.

[71] Herzig DO, Buie WD, Weiser MR, You YN, Rafferty JF, Feingold D, Steele SR (2017). Clinical practice guidelines for the surgical treatment of patients with Lynch syndrome. Dis Colon Rectum.

[72] Kalady MF, Lipman J, McGannon E, Church JM (2012). Risk of colonic neoplasia after proctectomy for rectal cancer in hereditary nonpolyposis colorectal cancer. Ann Surg.

[73] Win AK, Parry S, Parry B (2013). Risk of metachronous colon cancer following surgery for rectal cancer in mismatch repair gene mutation carriers. Ann Surg Oncol.

[74] (2025). American Liver Foundation: Primary sclerosing cholangitis (PSC). http://liverfoundation.org/liver-diseases/autoimmune-liver-diseases/primary-sclerosing-cholangitis-psc.

[75] Khaderi SA, Sussman NL (2015). Screening for malignancy in primary sclerosing cholangitis (PSC). Curr Gastroenterol Rep.

[76] Raza Z, Saqib SU, Bajwa AA (2024). Integrating artificial intelligence techniques for advancements in colorectal cancer management: navigating past and predicting future direction. J Pak Med Assoc.

[77] (2025). Role of AI in detection and management of colorectal polyps and cancer. http://mayoclinic.org/medical-professionals/digestive-diseases/news/role-of-ai-in-detection-and-management-of-colorectal-polyps-and-cancer.

[78] Wang P, Berzin TM, Glissen Brown JR (2019). Real-time automatic detection system increases colonoscopic polyp and adenoma detection rates: a prospective randomised controlled study. Gut.

[79] Huang D, Shen J, Hong J (2022). Effect of artificial intelligence-aided colonoscopy for adenoma and polyp detection: a meta-analysis of randomized clinical trials. Int J Colorectal Dis.

[80] Samarasena J, Yang D, Berzin TM (2023). AGA clinical practice update on the role of artificial intelligence in colon polyp diagnosis and management: commentary. Gastroenterology.

[81] Joseph J, LePage EM, Cheney CP, Pawa R (2021). Artificial intelligence in colonoscopy. World J Gastroenterol.

[82] Areia M, Mori Y, Correale L (2022). Cost-effectiveness of artificial intelligence for screening colonoscopy: a modelling study. Lancet Digit Health.

